# Phenformin enhances the therapeutic effect of selumetinib in KRAS-mutant non-small cell lung cancer irrespective of LKB1 status

**DOI:** 10.18632/oncotarget.19779

**Published:** 2017-08-01

**Authors:** Jun Zhang, Sreenivas Nannapaneni, Dongsheng Wang, Fakeng Liu, Xu Wang, Rui Jin, Xiuju Liu, Mohammad Aminur Rahman, Xianghong Peng, Guoqing Qian, Zhuo G. Chen, Kwok-Kin Wong, Fadlo R. Khuri, Wei Zhou, Dong M. Shin

**Affiliations:** ^1^ Department of Hematology and Medical Oncology, Emory University School of Medicine, Atlanta, GA 30322, USA; ^2^ Winship Cancer Institute, Emory University School of Medicine, Atlanta, GA 30322, USA; ^3^ Division of Hematology, Oncology and Blood & Marrow Transplantation, Department of Internal Medicine, Holden Comprehensive Cancer Center, University of Iowa Carver College of Medicine, Iowa City, IA 52242, USA; ^4^ Dana-Farber Cancer Institute, Harvard Medical School, Dana Building 810B, HIM243, Boston, MA 02115, USA

**Keywords:** NSCLC, KRAS, LKB1, phenformin, selumetinib

## Abstract

MEK inhibition is potentially valuable in targeting KRAS-mutant non-small cell lung cancer (NSCLC). Here, we analyzed whether concomitant LKB1 mutation alters sensitivity to the MEK inhibitor selumetinib, and whether the metabolism drug phenformin can enhance the therapeutic effect of selumetinib in isogenic cell lines with different LKB1 status. Isogenic pairs of KRAS-mutant NSCLC cell lines A549, H460 and H157, each with wild-type and null LKB1, as well as genetically engineered mouse-derived cell lines 634 (*kras*^G12D/wt^*/p53*^-/-^*/lkb1*^wt/wt^) and t2 (*kras*^G12D/wt^*/p53*^-/-^/*lkb1*^-/-^) were used *in vitro* to analyze the activities of selumetinib, phenformin and their combination. Synergy was measured and potential mechanisms investigated. The *in vitro* findings were then confirmed *in vivo* using xenograft models. The re-expression of wild type LKB1 increased phospho-ERK level, suggesting that restored dependency on MEK->ERK->MAPK signaling might have contributed to the enhanced sensitivity to selumetinib. In contrast, the loss of LKB1 sensitized cells to phenformin. At certain combination ratios, phenformin and selumetinib showed synergistic activity regardless of LKB1 status. Their combination reduced phospho-ERK and S6 levels and induced potent apoptosis, but was likely through different mechanisms in cells with different LKB1 status. Finally, in xenograft models bearing isogenic A549 cells, we confirmed that loss of LKB1 confers resistance to selumetinib, and phenformin significantly enhances the therapeutic effect of selumetinib. Irrespective of LKB1 status, phenformin may enhance the anti-tumor effect of selumetinib in KRAS-mutant NSCLC. The dual targeting of MEK and cancer metabolism may provide a useful strategy to treat this subset of lung cancer.

## INTRODUCTION

KRAS is one of the most commonly mutated oncogenes in NSCLC, with a reported frequency of 15–30% [1]. Due to the lack of a direct RAS inhibitor with clinically proven efficacy [2], targeting RAS downstream signaling such as the RAF->MEK->ERK->MAPK pathway has become a promising approach [3]. Since RAS activates more than a dozen downstream signaling pathways [4], a combination approach was found necessary to achieve durable response [5, 6]. In a recent large scale screening [7], MEK inhibitors were among the most effective agents in KRAS-mutant cancers [7], making MEK inhibitor a promising backbone for combination therapy [8].

In combination with docetaxel, the MEK inhibitor selumetinib was shown initially in a phase II trial to be highly promising in advanced NSCLC with KRAS mutation [9]. However, this initial signal of efficacy was not confirmed in a recent phase III study, the SELECT-1 trial (NCT01933932). One of the potential explanations is that concomitant mutations (e.g. PIK3CA, PTEN, LKB1, etc.) could modify the treatment response as shown previously [10, 11]. For example, in a study based on genetically-engineered mouse (GEM) models, concomitant loss of LKB1 conferred primary resistance to the combination of selumetinib with docetaxel [11]. Considering KRAS also activates multiple other signaling pathways [4], identifying novel MEK inhibitor-based combination therapies with potential effectiveness in cancer cells with various concomitant mutations is therefore urgently needed. An ideal combination would enhance the therapeutic effect of MEK inhibition (MEKi), have minimal or no added side effects, and be potentially effective against a wide range of concomitant mutations.

Targeting cancer metabolism in combination with MEK inhibition might be such a candidate strategy. Studies have shown that targeting cancer metabolism is effective in tumors harboring different mutations in either oncogenes (e.g. MYC, PIK3CA, etc.) or tumor suppressors (e.g. TP53, PTEN, LKB1, etc.) [12–14], likely because those genetic alterations are actively involved in the metabolic process and control the metabolic rewiring of cancer cells [15]. In fact, due to the unique metabolic features of cancer cells, genetic changes that confer resistance to traditional therapies may paradoxically offer treatment advantages when cancer metabolism is targeted [12, 16].

Phenformin, a biguanide antidiabetic agent is such a compound that inhibits cancer metabolism by primarily targeting mitochondria complex I [12]. Phenformin has been shown to have direct anti-cancer effects [17–19] and to enhance the therapeutic effects of either radiation [20] or targeted therapies [21]. While the loss of LKB1 promotes lung cancer metastasis [22], LKB1 also functions as a master regulator of cell metabolism through activation of the downstream AMPK signaling pathway [23]. With the presence of functional LKB1, the depletion of ATP by phenformin activates AMPK signaling which subsequently inhibits the mTOR pathway resulting in growth arrest [24]. However, with the loss of functional LKB1, the reduced ability to adapt to energy stress due to inactivation of AMPK signaling directly renders the cells prone to apoptosis [16, 24]. This dual effect of phenformin (and also metformin) under alternative LKB1 status makes its combination with MEKi an appealing strategy for KRAS-mutant NSCLC. In addition, lung cancer in general has high rate of aerobic glycolysis [25, 26] (hence the clinical use of PET imaging [27]), which becomes even more prominent in the setting of KRAS and LKB1 mutations [11], therefore making the use of phenformin even more relevant [16].

On the basis of these data, we aimed to determine if concomitant LKB1 mutation in KRAS-mutant NSCLC directly associates with decreased response to MEK inhibitor selumetinib, and if so, to define the underlying mechanism. We also assessed whether phenformin can overcome the resistance by enhancing the therapeutic effect of selumetinib. The findings from this study also help us to understand whether the dual targeting of MEK and cancer metabolism may be a novel and effective strategy to tackle KRAS-mutant NSCLC.

## RESULTS

### A systematic review suggests concomitant LKB1 mutation in KRAS-mutant NSCLC confers relative resistance to the MEK inhibitor selumetinib

**Figure 1:**
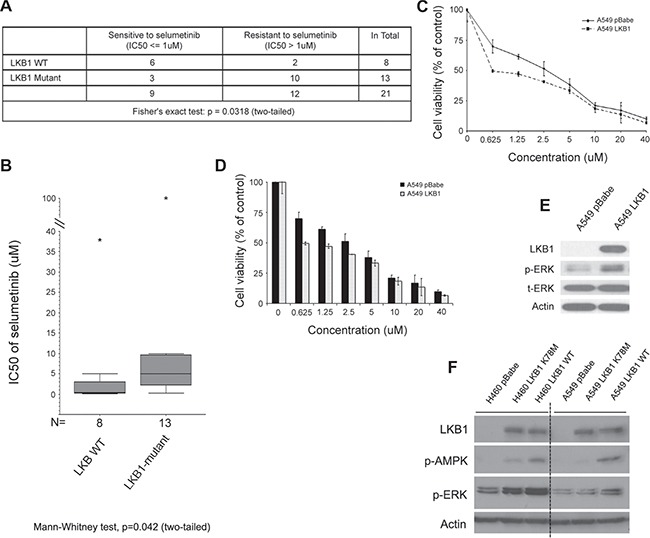
Concomitant LKB1 mutation correlates with selumetinib resistance and decreased level of p-ERK in KRAS-mutant NSCLC (**A**) Except the two cell lines (Calu-1 and H358) with controversial reported sensitivity to selumetinib, all other cell lines listed in Table 1 are included here for statistical analysis. When using IC50 > 1 μM to define resistance to selumetinib, cell lines with concomitant LKB1 mutation (excluding silent mutation) have significantly higher chance of resistance (Fisher›s exact test: *p* = 0.0318, two-tailed). (**B**) A direct comparison of IC50 between LKB1 wild type (including silent mutation) and mutant NSCLC cell lines. Whenever possible, for each cell line, the median value of reported IC50 was used for the Mann–Whitney nonparametric test. For cell lines only having a range of value, such as > x or < y μM, then x or y value was used for estimation (*p* = 0.042, two-tailed). The “*” stands for outliers. (**C**) Representative growth inhibition assay. Isogenic A549^pBabe^ and A549^LKB1^ cells were incubated with different concentrations of selumetinib for 72 hrs. With the re-expression of LKB1, cells were more sensitive to selumetinib with lower IC50. (**D**) Histogram of c. Cells were tested in quadruplicates. (**E**) Loss of LKB1 in A549^pBabe^ cells was associated with low level of p-ERK, suggesting decreased dependency on MEK->ERK->MAPK signaling. (**F**) A similar phenomenon was observed using other isogenic cell lines. When using cells engineered with kinase dead LKB1 (K78M) as comparison, fully functional wild type LKB1 had the most definitive association with elevated level of p-ERK.

Although a previous study based on GEM models suggested that concomitant *lkb1* loss in the setting of *kras*^G12D^ mutation confers primary resistance to selumetinib and docetaxel in combination [11], it did not provide clear evidence to show decreased sensitivity to selumetinib alone, probably due to small sample size (their Supplementary Figure 5) [11]. To address this question, we first conducted a systematic review aiming to identify all NSCLC cell lines harboring *KRAS* mutation that were tested with selumetinib in the literature. Table 1 is the summary of these 23 cell lines with their *KRAS* and *LKB1* status. When we used IC50 < = 1 μM to define the sensitive cell lines and > 1 μM for the resistant ones, we observed a correlation between concomitant *LKB1* mutation and relative resistance to selumetinib (Figure 1A, two-tailed Fisher's exact test, *p* = 0.0318). Due to the contradictory reports regarding Calu-1 and H358 cells in the literature, only 21 out of the 23 cell lines were used for statistical analysis. Since the H1155 cell line has a silent *LKB1* mutation, it was included in the LKB1 wild type group. An attempt to compare the reported IC50 value by using nonparametric Mann–Whitney test also revealed concomitant *LKB1* mutation correlates with higher IC50 (Figure 1B, two-tailed, *p* = 0.042). Interestingly, when we expanded the criteria to include NSCLC cell lines harboring any RAS and/or RAF mutations, we observed an even stronger correlation possibly due to the increased sample size (Supplementary Table 1 and Supplementary Figure 1A and 1B).

**Table 1:** Characterization of the 23 NSCLC cell lines used in the systematic review

Cell line	KRAS status	LKB1 status	Selumetinib IC50 (μM)
Considered as sensitive to selumetinib (IC50 < = 1 mM)			
H441	KRAS G12V	WT^(1)^	< 0.30^(2)^
Calu-6	KRAS Q61K	WT^(3)^	0.32^(4)^, 0.33^(2)^, 1.0^(5)^
SK-LU-1	KRAS G12D	WT^(3)^	0.5^(5)^
H2009	KRAS G12A	WT^(6, 7)^	0.99^(4)^
H727	KRAS G12V	WT^(1)^	0.01^(8)^
SW900	KRAS G12V	WT^(9)^	0.28^(8)^
H1944	KRAS G13D	K62N, K78N	< 0.30^(2)^
A427	KRAS G12D	Null^(10, 11)^	0.55^(2)^
H2122	KRAS G12C	P281fs*6, deletion^(10, 12)^	< 0.1^(5)^, 1.0^(13)^
Considered as resistant to selumetinib (IC50 > 1 μM)			
A549	KRAS G12S	Q37*	0.8^(2)^, > 1^(14)^, 5^(15)^, ~5^(16)^, 6.3^(8)^, ~10^(5)^, > 10^(17)^
H23	KRAS G12C	W332*	1.5^(2)^, > 10^(17)^
H460	KRAS G12S	Q37*	1.7^(2)^, 9.6^(8)^, > 10^(15)^, > 10^(5)^
H2030	KRAS G12C	E317*, E357K, M392I	2.2^(2)^
H2122	KRAS G12C	P281fs*6	~3^(16)^
H1734	KRAS G13C	M51fs*14	4.2^(2)^
H157	KRAS G12R	Null^(10, 11)^	9.3^(8)^, > 10^(17)^
HCC44	KRAS G12C	M51I, 52 → 162 stop^(10)^	~10^(16)^
H1355	KRAS G13C	R49L^(6, 18)^	~100^(16)^
H647	KRAS G13D	Null^(19)^	> 5.0^(2)^, > 10^(5)^
H2887	KRAS G12V^(6, 20, 21)^	WT^(6)^	38^(16)^
H1155	KRAS Q61H	WT (silent: I46I, P281P)	> 5.0^(2)^
Controversial results in literature			
Calu-1	KRAS G12C	WT^(12)^	< 0.2^(2)^, > 1^(14)^, ~130^(16)^
H358	KRAS G12C	WT^(12)^	0.2^(17)^, 0.5^(2)^, 1.0^(14)^, ~10^(16)^, > 10^(8)^

All cell lines were extracted from the literature according to the search criteria stated in the text. They all have KRAS mutation, and were tested with selumetinib. Unless specifically noted, all mutation profiles were confirmed in COSMIC database. Since COSMIC database does not report wild type (WT) genes, the wild type LKB1 status was confirmed through literature search. Numbers in parentheses correspond to the cited studies.

**References**

1. Molina-Arcas M, Hancock DC, Sheridan C, Kumar MS, Downward J. Coordinate direct input of both KRAS and IGF1 receptor to activation of PI3 kinase in KRAS-mutant lung cancer. Cancer Discov. 2013; 3:548–63.

2. Garon EB, Finn RS, Hosmer W, Dering J, Ginther C, Adhami S, et al. Identification of common predictive markers of *in vitro* response to the Mek inhibitor selumetinib (AZD6244; ARRY-142886) in human breast cancer and non-small cell lung cancer cell lines. Mol Cancer Ther. 2010; 9:1985–94.

3. Onozato R, Kosaka T, Achiwa H, Kuwano H, Takahashi T, Yatabe Y, et al. LKB1 gene mutations in Japanese lung cancer patients. Cancer Sci. 2007; 98:1747–51.

4. Meng J, Peng H, Dai B, Guo W, Wang L, Ji L, et al. High level of AKT activity is associated with resistance to MEK inhibitor AZD6244 (ARRY-142886). Cancer Biol Ther. 2009; 8:2073–80.

5. Dry JR, Pavey S, Pratilas CA, Harbron C, Runswick S, Hodgson D, et al. Transcriptional pathway signatures predict MEK addiction and response to selumetinib (AZD6244). Cancer Res. 2010; 70:2264–73.

6. Greer RM, Peyton M, Larsen JE, Girard L, Xie Y, Gazdar AF, et al. SMAC mimetic (JP1201) sensitizes non-small cell lung cancers to multiple chemotherapy agents in an IAP-dependent but TNF-α-independent manner. Cancer Res. 2011; 71:7640–8.

7. Mahoney CL, Choudhury B, Davies H, Edkins S, Greenman C, Haaften G, et al. LKB1/KRAS mutant lung cancers constitute a genetic subset of NSCLC with increased sensitivity to MAPK and mTOR signalling inhibition. Br J Cancer. 2009; 100:370–5.

8. Friday BB, Yu C, Dy GK, Smith PD, Wang L, Thibodeau SN, et al. BRAF V600E disrupts AZD6244-induced abrogation of negative feedback pathways between extracellular signal-regulated kinase and Raf proteins. Cancer Res. 2008; 68:6145–53.

9. Hata AN, Yeo A, Faber AC, Lifshits E, Chen Z, Cheng KA, et al. Failure to induce apoptosis via BCL-2 family proteins underlies lack of efficacy of combined MEK and PI3K inhibitors for KRAS mutant lung cancers. Cancer Res. 2014; 74:3146–56.

10. Matsumoto S, Iwakawa R, Takahashi K, Kohno T, Nakanishi Y, Matsuno Y, et al. Prevalence and specificity of LKB1 genetic alterations in lung cancers. Oncogene. 2007; 26:5911–8.

11. Shackelford DB, Abt E, Gerken L, Vasquez DS, Seki A, Leblanc M, et al. LKB1 inactivation dictates therapeutic response of non-small cell lung cancer to the metabolism drug phenformin. Cancer Cell. 2013; 23:143–58.

12. Liu Y, Marks K, Cowley GS, Carretero J, Liu Q, Nieland TJ, et al. Metabolic and functional genomic studies identify deoxythymidylate kinase as a target in LKB1-mutant lung cancer. Cancer Discov. 2013; 3:870–9.

13. Sun C, Hobor S, Bertotti A, Zecchin D, Huang S, Galimi F, et al. Intrinsic resistance to MEK inhibition in KRAS mutant lung and colon cancer through transcriptional induction of ERBB3. Cell Rep. 2014; 7:86–93.

14. Acquaviva J, Smith DL, Sang J, Friedland JC, He S, Sequeira M, et al. Targeting KRAS-mutant non-small cell lung cancer with the Hsp90 inhibitor ganetespib. Mol Cancer Ther. 2012; 11:2633–43.

15. Troiani T, Vecchione L, Martinelli E, Capasso A, Costantino S, Ciuffreda LP, et al. Intrinsic resistance to selumetinib, a selective inhibitor of MEK1/2, by cAMP-dependent protein kinase A activation in human lung and colorectal cancer cells. Br J Cancer. 2012; 106:1648–59.

16. Meng J, Dai B, Fang B, Bekele BN, Bornmann WG, Sun D, et al. Combination treatment with MEK and AKT inhibitors is more effective than each drug alone in human non-small cell lung cancer *in vitro* and *in vivo*. PloS one. 2010; 5:e14124.

17. Yoon YK, Kim HP, Han SW, Oh dY, Im SA, Bang YJ, et al. KRAS mutant lung cancer cells are differentially responsive to MEK inhibitor due to AKT or STAT3 activation: implication for combinatorial approach. Mol Carcinog. 2010; 49:353–62.

18. Tsai LH, Chen PM, Cheng YW, Chen CY, Sheu GT, Wu TC, et al. LKB1 loss by alteration of the NKX2-1/p53 pathway promotes tumor malignancy and predicts poor survival and relapse in lung adenocarcinomas. Oncogene. 2013.

19. Wallin JJ, Edgar KA, Guan J, Berry M, Prior WW, Lee L, et al. GDC-0980 is a novel class I PI3K/mTOR kinase inhibitor with robust activity in cancer models driven by the PI3K pathway. Mol Cancer Ther. 2011; 10:2426–36.

20. Querings S, Altmüller J, Ansén S, Zander T, Seidel D, Gabler F, et al. Benchmarking of mutation diagnostics in clinical lung cancer specimens. PloS one. 2011; 6:e19601.

21. Barbie DA, Tamayo P, Boehm JS, Kim SY, Moody SE, Dunn IF, et al. Systematic RNA interference reveals that oncogenic KRAS-driven cancers require TBK1. Nature. 2009; 462:108–12.

### LKB1 inactivation associates with decreased sensitivity to selumetinib and reduced phospho-ERK level in isogenic KRAS-mutant NSCLC cell lines

To confirm the findings from our systematic review, we used isogenic *KRAS*-mutant NSCLC cell lines to study how *LKB1* affects the response to selumetinib in the setting of *KRAS* mutation. Using the pBABEpuro-based retroviral infection system, we established isogenic A549, H460 and H157 stable cell lines over-expressing wild type LKB1 (labeled A549^LKB1^, H460^LKB1^ and H157^LKB1^ respectively) compared to their clones infected with empty vector (named A549^pBabe^, H460^pBabe^ and H157^pBabe^ respectively). Shown as an example in Figure 1C and 1D, A549^LKB1^ cells were more sensitive to selumetinib at certain concentrations than A549^pBabe^ cells. A similar effect was observed in isogenic H460 and H157 cell lines (Supplementary Figure 1C and 1D). When exploring possible mechanisms for this observation, we found that A549^pBabe^ cells have a very low level of phospho-ERK1/2 (p-ERK1/2) compared to A549^LKB1^ cells (Figure 1E), suggesting LKB1 inactivation is associated with less dependency on the MEK->ERK->MAPK signaling pathway, and hence decreased sensitivity to the MEK inhibitor selumetinib. Re-expression of LKB1 significantly enhanced p-ERK1/2, suggesting increased dependency might be the potential reason for enhanced sensitivity to the inhibition of this signaling pathway. This is in agreement with the observation by Chen Z et al. of a significantly decreased p-ERK1/2 level in tumors of *kras*^G12D^/*lkb1*^-/-^ GEM mice compared to *kras*^G12D^/*lkb1*^+/+^ GEM mice by either IHC or Western blot, which correlated with the primary resistance to selumetinib and docetaxel combination therapy [11]. Interestingly, we observed the same phenomenon in other isogenic cell lines such as H460 (Figure 1F). When we used their isogenic cells expressing kinase dead LKB1 (K78M) as a comparison, we found that while fully functional wild type LKB1 was associated with arguably the highest level of p-ERK, the kinase dead LKB1 also led to an increased level of p-ERK. This suggests that loss of LKB1 protein via genetic alteration is critical in the reduction of p-ERK level, but LKB1 kinase activity is dispensable in this process. However, how LKB1 affects p-ERK, and whether there is a feedback loop sent from LKB1 to MAPK signaling since LKB1 can be negatively phosphorylated by ERK [28], are still currently under investigation.

### LKB1 inactivation dictates enhanced sensitivity to the metabolic drug phenformin, which may synergize with the antitumor effect of selumetinib in KRAS-mutant NSCLC cell lines

**Figure 2:**
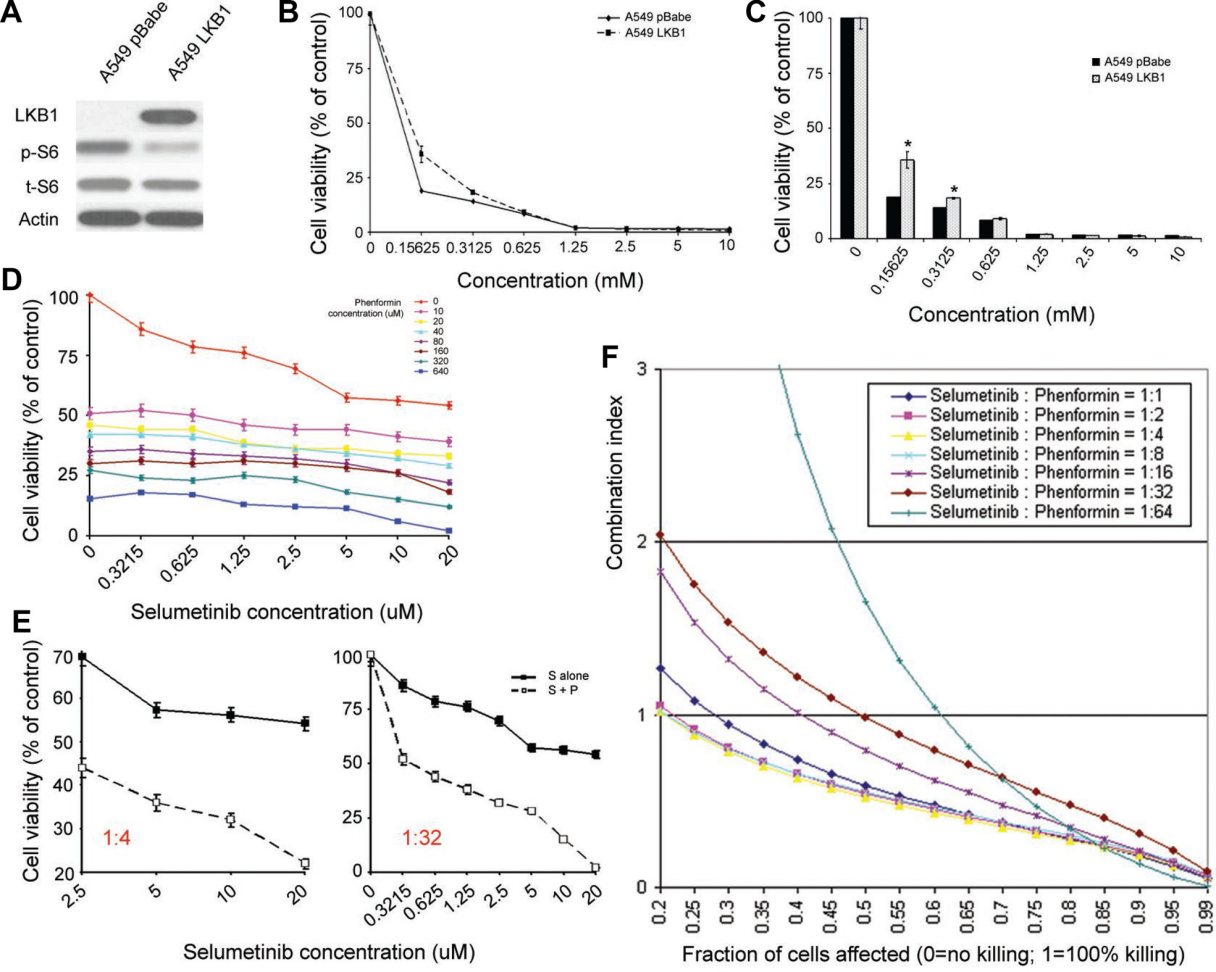
LKB1 inactivation dictates enhanced sensitivity to the metabolic drug phenformin, which enhances the antitumor effect of selumetinib in KRAS-mutant NSCLC cell lines (**A**) In A549 cells as an example, the upregulation of p-S6 was consistently observed in cells with LKB1 inactivation. (**B** and **C**) Growth inhibition assay. Shown here are the isogenic A549 cells with alternative LKB1 status. The loss of LKB1 rendered A549 cells more sensitive to phenformin. c is the histogram of b. (**D**) Cell proliferation assay using different concentrations and ratios of selumetinib and phenformin in combination. The experiment ended at ~ 40 hrs after incubation. Cells were prepared in triplicate. (**E**) An illustration to show that under certain combination ratio (e.g. selumetinib/phenformin=1:4 or 1:32), phenformin enhanced the antitumor effect of selumetinib in ~ 40 hrs. (**F**) CalcuSyn was used to calculate the combination index, and demonstrated a synergistic effect at certain concentration and combination of selumetinib and phenformin. A549^pBabe^ cells were used in d, e & f. For similar studies using A549^LKB1^ cells, please refer to the Supplementary Figure 2C and 2E. S: selumetinib; P: phenformin; S+P: selumetinib in combination with phenformin. The asterisks (*) denote statistical significance (*p* < 0.01).

In order to identify a potential MEKi based combination therapy to circumvent selumetinib resistance relating to LKB1 mutation, we performed an initial screening to examine the major differences between the isogenic cells. Using A549 cells as an example, we found consistent elevation of p-S6 in A549^pBabe^ cells compared to A549^LKB1^ cells (Figure 2A), suggesting that a combination with mTOR inhibitor such as AZD8055 might be fruitful. However, a pilot study did not demonstrate clear selectivity of AZD8055 for LKB1-mutamt cells (Supplementary Figure 2A), possibly because AZD8055 inhibits both mTORC1 and mTORC2 while the inhibitory effect on mTOR from LKB1-AMPK activation is primarily on mTORC1 [29].

LKB1 is an energy sensor, and it has been recently shown in NSCLC that regardless of the genetic background, LKB1 inactivation dictates enhanced sensitivity to the metabolic drug phenformin [16]. Thus, we wondered if we could circumvent selumetinib resistance associated with LKB1 loss by combining with phenformin (Supplementary Figure 4). In addition, since phenformin may serve as an AMPK activator in cells with wild type LKB1 [16, 21, 24], and negatively regulates mTOR activity, it is also reasonable to combine selumetinib with phenformin in cancer cells with wild type LKB1 (Supplementary Figure 4). More importantly, this combination could serve as a proof-of-concept study to investigate the value of dual targeting MEK and cancer metabolism for KRAS-mutant NSCLC.

Using isogenic A549 cells as an example, Figure 2B and 2C shows that A549^pBabe^ cells were more sensitive to phenformin than A549^LKB1^ cells, which is consistent with reported findings [16], although a much lower dose was used in our proliferation assay. When we tested phenformin in cell lines established from GEM models, namely 634 (*kras*^G12D/wt^/*p53*^-/-^/*lkb1*^wt/wt^) and t2 (*kras*^G12D/wt^/*p53*^-/-^/*lkb1*^-/-^) [30], we observed a similar phenomenon (Supplementary Figure 2B). We then tested the combination effect of selumetinib and phenformin with different concentration ratios in A549 cells with alternative LKB1 status (Figure 2D and Supplementary Figure 2C), and found that at certain concentration range and combination ratios, phenformin enhanced the effect of selumetinib irrespective of LKB1 status (Figure 2E and Supplementary Figure 2D). When we used CalcuSyn to calculate the combination index, we observed a synergistic effect at certain concentration ratios irrespective of LKB1 status, although the optimal ratio between selumetinib and phenformin was different (Figure 2F and Supplementary Figure 2E). This is likely due to different sensitivity to selumetinib and phenformin in cells with alternative LKB1 status.

### Phenformin enhances the anti-tumor effect of selumetinib *in vitro* through different mechanisms in KRAS-mutant NSCLC cell lines with alternative LKB1 status

**Figure 3:**
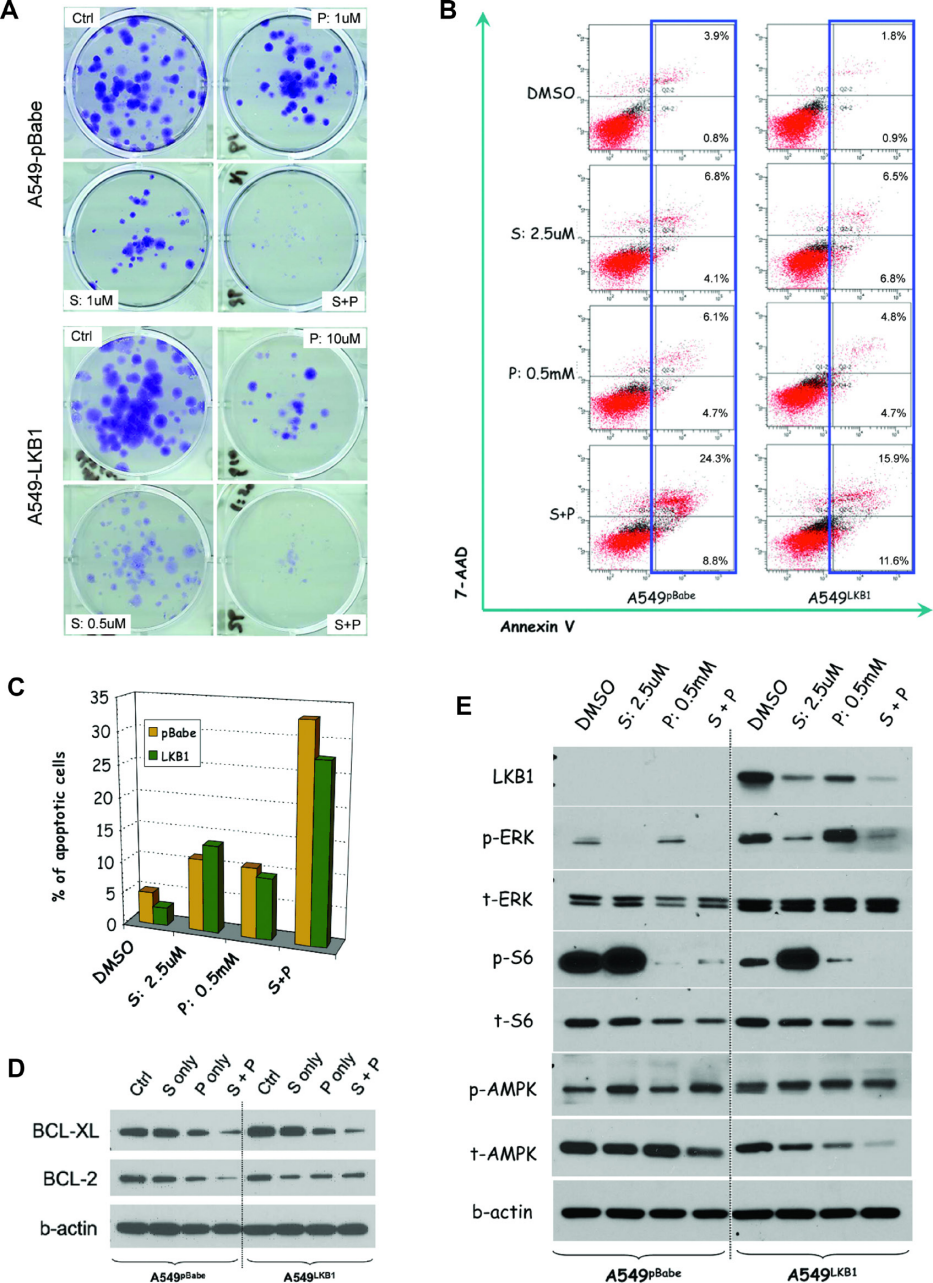
Phenformin enhances the anti-tumor effect of selumetinib *in vitro* through different mechanisms in KRAS-mutant NSCLC cell lines with alternative LKB1 status (**A**) Colony assays starting with 200 cells after incubation with DMSO (ctrl), selumetinib (S), phenformin (P) or the combination (S+P) for 2 weeks. Regardless of the LKB1 status, the combination of S and P had better growth inhibition effect than either agent alone. Please note the different ratios of S and P used for each cell line. (**B**) After 48 hours treatment, the apoptotic population was measured via flow cytometry based on 7-AAD and annexin V staining. Irrespective of LKB1 status, the combination treatment resulted in more apoptotic cells. (**C**) Histogram representation of b. (**D**) In both A549^pBabe^and A549^LKB1^ cells, the combination therapy potently downregulated BCL-XL level. However, only in A549^pBabe^ cells, the S+P combination reduced BCL-2 level more significantly than either S or P alone. (**E**) Western blot showing LKB1 inactivation resulted in lower level of p-ERK but high p-S6. Selumetinib alone potently suppressed p-ERK but upregulated p-S6 after incubation for 48 hrs. Phenformin helped suppress p-S6. Although in A549^LKB1^ cells, the suppression was parallel to AMPK activation (i.e. increased p-AMPK/t-AMPK ratio), in A549^pBabe^ cells, no significant change in p-AMPK/t-AMPK ratio was observed.

To confirm the potential synergism, a colony assay was performed, which again demonstrated that at certain combination ratios, phenformin could significantly enhance the anti-tumor effect of selumetinib (Figure 3A). Similar results were observed in H460 isogenic cells as well (Supplementary Figure 5A). Since apoptosis is one of the most important mechanisms of cell death, we investigated the effect of combination treatment on cell apoptosis. By using flow cytometry to quantify the apoptotic population after 48 hours of treatment, we found the combination of phenformin and selumetinib resulted in significantly more apoptotic cells irrespective of LKB1 status (Figure 3B and 3C). This observation correlated well with significant down-regulation of the anti-apoptotic protein BCL-XL, which is abundantly expressed in lung cancers and correlates with poor prognosis [31, 32] (Figure 3D). Interestingly, with unknown mechanism, a significantly reduced level of BCL-2 after combination treatment was also observed in A549^pBabe^ cells but not in A549^LKB1^ cells (Figure 3D).

Since phenformin may inhibit mTOR signaling through the activation of AMPK in cells with wild type LKB1 [16, 21, 24], but the loss of LKB1 dictates sensitivity to phenformin [16], we wondered if the observed synergy was due to different mechanisms in cells with different LKB1 status. An analysis of potential involved signaling pathways showed in both cases, the combination of phenformin and selumetinib significantly inhibited p-ERK and p-S6 (Figure 3E). In A549^pBabe^ cells, compared to the control group receiving DMSO, phenformin did not affect the p-AMPK/t-AMPK (total AMPK) ratio. However, in A549^LKB1^ cells, there was significant elevation of p-AMPK/t-AMPK ratio, suggesting the inhibition of p-S6 by phenformin in A549^LKB1^ cells was parallel to the activation of AMPK but was AMPK-independent in A549^pBabe^ cells (Figure 3E). To confirm above findings are not A549 cell line specific, we performed similar experiments using isogenic H460 cells. As shown in Supplementary Figure 5C, the combination of selumetinib and phenformin again significantly reduced the levels of p-ERK and p-S6. Consistent to our observation from A549 isogenic cells, only in H460^LKB1^ cells, such inhibition of phosphorylated ERK and S6 was parallel to the activation of AMPK. Taking these data together, although phenformin enhanced the effect of selumetinib regardless of the LKB1 status, the underlying mechanisms were different.

### Phenformin enhances the therapeutic effect of selumetinib *in vivo* regardless of the LKB1 status, and their combination resulted in more robust apoptosis and attenuation of major growth signaling pathways

**Figure 4:**
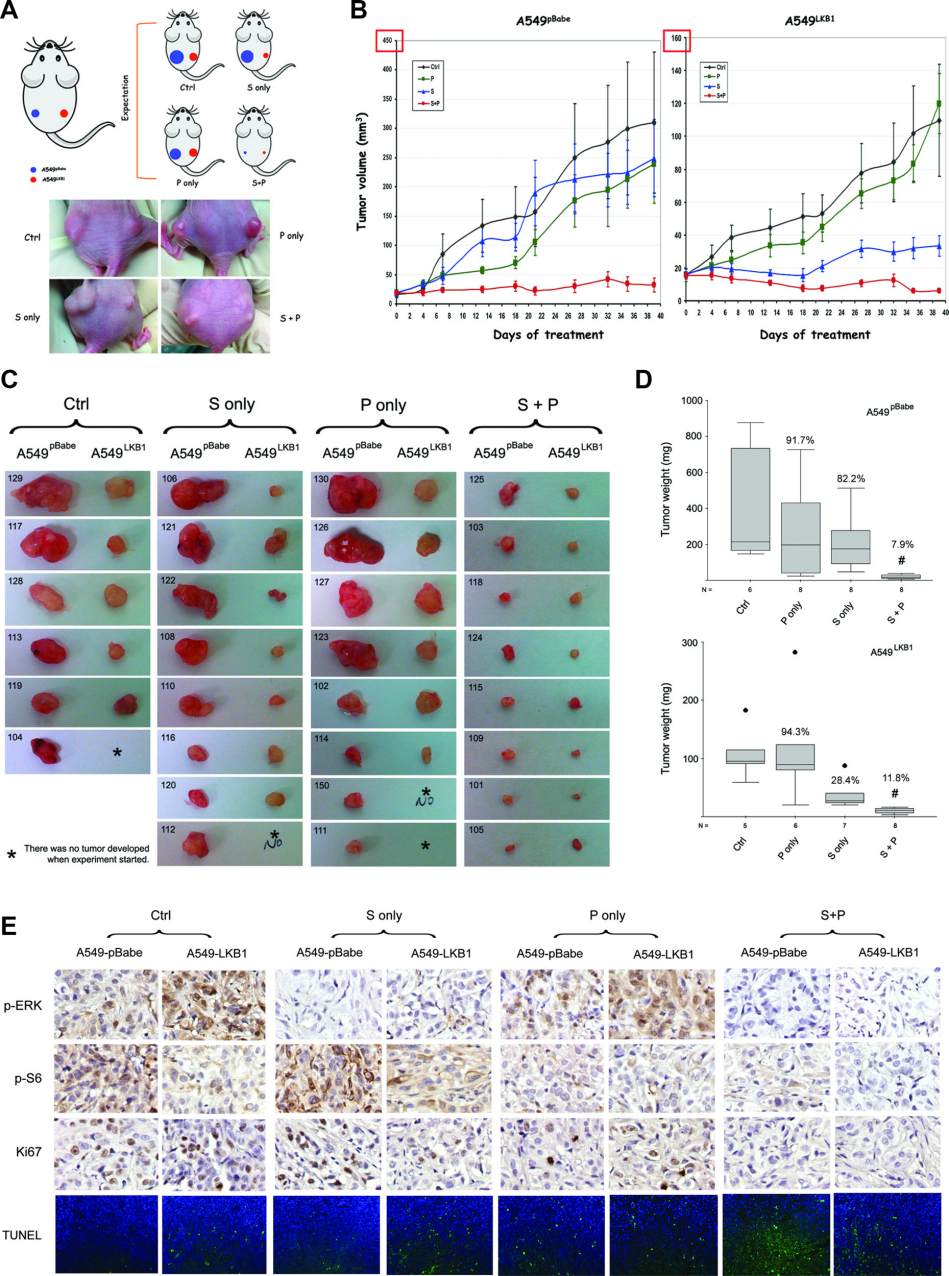
Phenformin enhances the therapeutic effect of selumetinib *in vivo* regardless of LKB1 status (**A**) Upper panel: illustration of the xenograft model in nude mice. ~ 1.5 million A549^pBabe^ and A549^LKB1^ cells were implanted on the left and right flank of nude mice respectively. Treatment started once the tumors became palpable. Mice were orally gavaged daily with DMSO (ctrl), selumetinib (S, 50 mg/kg), phenformin (P, 100 mg/kg) or the combination (S+P), 5 days per week. Lower panel: representative mouse from each group on day 26 post-treatment. (**B**) Xenograft tumor growth curve. A549^LKB1^ tumors were smaller than A549^pBabe^ tumors, consistent with the tumor-suppressing function of LKB1. A549^pBabe^ tumors were resistant to selumetinib whereas A549^LKB1^ tumors were sensitive, consistent with *in vitro* data shown in the previous figures. Although A549^pBabe^ tumors were more sensitive to phenformin initially, growth of some tumors quickly caught up resulting in no statistical difference after day 18. The combination of S and P potently inhibited the growth of tumors of both A549^pBabe^ and A549^LKB1^ cells. (**C**) All tumors harvested from the mice. Tumors of A549^pBabe^ and A549^LKB1^ cells from the same mice were placed next to each other. Some mice did not develop tumor from A549^LKB1^ cells (labeled with *). The individual number indicates each individual mouse. The combination of selumetinib and phenformin demonstrated potent inhibition. (**D**) Box-and-Whisker plots showing the weight of tumors in c. The medians of tumor weight from each group were compared using nonparametric Kruskal–Wallis test. Tumors treated with combination therapy had the lowest weight (i.e. smallest). The percentage of median tumor weight over the control is shown on each Box-and-Whisker plot. (**E**) Representative IHC staining of p-ERK, p-S6, Ki67, and representative TUNEL staining of tumor sections from different groups with alternative LKB1 status. Again, the combination of selumetinib and phenformin resulted in the lowest signals of p-ERK, p-S6, Ki67 and TUNEL staining. The # denotes statistical significance (*p* < 0.01 in either case).

To directly compare treatment response in tumors with different LKB1 status, the isogenic A549^pBabe^ and A549^LKB1^ cells were implanted in the same mouse on the left and right flank respectively (Figure 4A upper panel). Our expectation was that if LKB1 does have tumor suppressing function, then the tumor on the right side (from A549^LKB1^ cells) would be smaller over time than the tumor on the left (from A549^pBabe^ cells). If the loss of LKB1 confers resistance to selumetinib, but dictates sensitivity to phenformin, then after selumetinib treatment the size difference between A549^pBabe^ and A549^LKB1^ tumors would be even greater. However, the difference would be smaller after treatment with phenformin. If phenformin enhances the anti-tumor effect of selumetinib, then tumors of both sides after combination treatment would be significantly smaller than control tumors or those treated with either selumetinib or phenformin alone (Figure 4A upper panel). As expected, A549^LKB1^ tumors (right) did grow more slowly than A549^pBabe^ tumors (left) in the control group (received diluted DMSO), and the loss of LKB1 (A549^pBabe^ tumors) did confer resistance to selumetinib (Figure 4B–4D, and Supplementary Figure 6). However, although the A549^pBabe^ tumors (left) were significantly smaller in mice treated with phenformin than in control mice before treatment day 18 (Figure 4B), some of these tumors eventually caught up and there was no significant difference on the day of sacrifice (Figure 4B–4D, and Supplementary Figure 6). This observation suggested that, at least in this xenograft model, single agent phenformin at the current dose/frequency was not sufficient to achieve long-term tumor suppression. However, phenformin did enhance the therapeutic effect of selumetinib significantly and consistently regardless of the LKB1 status (Figure 4B–4D). Importantly, at the dose used, all mice tolerated the combination treatment well without significant side effects observed (e.g. skin rash, body weight, gross histology of major organs, etc) (Supplementary Figure 3A and 3B).

IHC analysis of the tumor tissue confirmed that the combination of phenformin and selumetinib resulted in decreased levels of p-ERK regardless of LKB1 status (Figure 4E). In accordance with the enhanced apoptosis observed *in vitro* (Figure 3B–3D) and suppressed tumor growth *in vivo* (Figure 4B–4D), we also observed less Ki-67 and increased TUNEL signals in the group receiving combination treatment (Figure 4E). Hence, this combination treatment suppressed tumor growth likely through the inhibition of cell proliferation as well as the activation of apoptosis. When comparing the matched tumors in each group, A549^LKB1^ tumors showed significantly higher TUNEL signal than A549^pBabe^ tumors in the control group, which confirms the tumor suppressing function of LKB1 (Supplementary Figure 3C). In almost every mouse treated with selumetinib only, the TUNEL signal was significantly higher in the A549^LKB1^ tumor than its matched A549^pBabe^ tumor, consistent with our observation that the loss of LKB1 confers resistance to selumetinib whereas the restoration of wild type LKB1 enhances sensitivity (Supplementary Figure 3D). In the larger A549^pBabe^ tumors in the phenformin only group (e.g. #130, 126, 127 and 123 in Figure 4C), we observed only minimal TUNEL signal (data not shown), which could explain the loss of growth inhibition by phenformin at that time point (Figure 4B and 4D). A similar statistical analysis could not be performed in the combination (S+P) group due to tiny or even undetectable tumor foci.

## DISCUSSION

Through a meta-analysis based on multiple reported studies, as well as our own isogenic cell lines, we have clearly demonstrated that in the setting of *KRAS* mutation, LKB1 inactivation correlates with decreased response to the MEK inhibitor selumetinib probably through decreased dependency on MEK->ERK->MAPK signaling pathway. This is consistent with the previous observation using GEM models harboring *kras* G12D mutation [11]. Interestingly, using BRAF^V600E^ mutant melanoma cells, Zheng et al. demonstrated that LKB1 could be negatively phosphorylated at the Ser325 and Ser428 sites by ERK and RSK, respectively [28]. We thus wondered if a similar situation occurs in KRAS-mutant NSCLC, and whether there exists a negative feedback loop sent from LKB1 to RAF->MEK->ERK so that when LKB1 is inactivated, the activity of MAPK signaling is correspondingly attenuated, hence the seemingly reduced dependency. However, a recent study by Kaufman et al. using an LKB1 loss-associated gene expression signature suggested that human lung cancer differs substantially from the expression profile of the *kras*^G12D^/*lkb1* floxed GEM model [33], therefore raising the concern that the sensitivity to MEK inhibition observed in GEM models may not be extrapolated to human lung cancer with LKB1 mutation. While biological differences do exist between murine models and cancer patients, we found the gene expression profiling of human cancer in Kaufman's study has no selection regarding RAS/RAF status, but this was compared directly to the data derived from the *kras*^G12D^/*lkb1* floxed GEM which uniformly harbors *kras*^G12D^ mutation [33]. Therefore data needs to be interpreted cautiously, and preferably under a defined genetic context. This is especially important for LKB1 since its regulation and function is highly context dependent [34, 35].

Our study again confirmed that LKB1 inactivation sensitized lung cancer cells to phenformin as previously observed [16]. However, despite using comparable dose and frequency, in our xenograft model, phenformin alone was not sufficient to continue suppressing the growth even of tumors derived from LKB1-deficient A549^pBabe^ cells. This differs from the observations using GEM models, which demonstrated that phenformin alone was potent enough to significantly reduce lung tumor load in *kras/lkb1* double mutant mice [16]. These different outcomes may be due to differences in the metabolism of cancer cells modified by growth under different environments (e.g. flank vs. lungs), for example, subcutaneous implantation will likely place the cancer cells in a much less vascularized and more hypoxic environment compared to orthotopic growth [36]. Another factor may be suboptimal delivery of phenformin in our xenograft model. Nevertheless, it seems the tested concentrations of phenformin were sufficient to enhance the therapeutic response to selumetinib.

Interestingly, the enhancement of selumetinib activity by phenformin occurred regardless of LKB1 status, although different mechanisms were employed. In LKB1-wild type NSCLCs, the combination of MEK inhibitor and metformin was found to down-regulate GLI1 transcriptional activity to mediate an anti-tumor activity [37]. The action of phenformin paralleled the activation of AMPK in cells with wild type LKB1 but not in LKB1-deficient isogenic cells, consistent with the literature [16, 21]. However, in LKB1-deficient cells, whether phenformin suppressed p-S6 in an AMPK-independent manner through inhibiting mTORC1 in a rag GTPase-dependent manner much like metformin [38], or through other mechanisms needs further investigation. While the combination of selumetinib and phenformin significantly suppressed BCL-XL level regardless of LKB1 status, it is intriguing that BCL-2 was only significantly down-regulated *in vitro* in A549^pBabe^ cells. Although this could be due to different regulation of BCL-XL and BCL-2 as previously pointed out [39], the exact mechanism needs to be further explored. Nevertheless, the observation that phenformin was able to enhance selumetinib regardless of LKB1 status is important and novel. It suggests a pre-screening of LKB1 status is not necessary for this combination therapy. Since there is no standard approach to evaluate the functional status of LKB1 in the clinical setting (as it can be potentially affected by both genetic and epigenetic modifications), this finding offers convenience if such combination therapy is considered for KRAS-mutant NSCLC.

As a biguanide anti-diabetic drug, phenformin was withdrawn from the market in the 1970s due to rare but severe lactic acidosis [40, 41]. However, it has several advantages over its sister drug metformin from the perspective of targeting cancer metabolism: 1) it is almost 50 times more potent than metformin in targeting mitochondrial complex I [42]; 2) it does not require specific transporters as metformin does, therefore it has superior bioavailability than metformin [43]. Because of these features, there is resurged interest in using phenformin for cancer treatment, and side effects are expected to be less common in cancer therapy since the agent does not need to be taken daily as in diabetes treatment [16]. However, with the current unavailability of phenformin for clinical use, it will be interesting to test MEK inhibition in combination with metformin, especially considering metformin has anti-cancer mechanisms specifically relevant to KRAS-mutant cancer, such as down-regulation of PI3K->AKT->mTOR signaling [44], displacement of constitutively active KRAS from the cell membrane and uncoupling of the MAPK signaling pathway [45]. This idea is supported by a recent published study by Vujic et al. who showed metformin synergized with trametinib (another MEK inhibitor) in NRAS-mutant melanoma, lung cancer and neuroblastoma [46]. In fact, we have designed a similar murine lung cancer co-clinical trial as previously reported [11], to combine metformin and MEK inhibition in KRAS-driven lung cancer GEM models with different concomitant mutations, and plan to incorporate the data into a clinical trial using the same combination for patients with KRAS-mutant NSCLC.

Overall, our study has not only identified a novel combination of phenformin with MEK inhibition for KRAS-mutant NSCLC, it also provides proof of concept that dual targeting of an oncogenic growth signal and cancer metabolism can be a novel and fruitful approach to achieve steady and significant tumor suppression. However, since the metabolic rewiring of cancer cells is very context dependent [12], mitochondrial complex 1 may not always be the best target. Therefore, one of our future goals is to identify the potential metabolism targets in a defined genetic and phenotypic context, for example, to identify the most important enzyme or metabolic intermediate in the most crucial metabolic pathway (e.g. glycolysis vs. TCA cycle vs. glutaminolysis, etc.) in lung adenocarcinoma harboring KRAS mutation, and determine how concomitant genetic alterations such as LKB1 inactivation modify the metabolic targets. Such investigations have the potential to make targeting cancer metabolism more precise, and to add another layer of accuracy in personalized therapy.

In summary, our study has demonstrated that in KRAS-mutant NSCLC, concomitant LKB1 mutation correlates with decreased response to selumetinib. However, regardless of LKB1 status, phenformin enhances the therapeutic response of selumetinib. This study serves as the proof of concept that dual targeting of MEK and cancer metabolism may be a novel approach to tackle KRAS-mutant NSCLC.

## MATERIALS AND METHODS

### Systematic review

The search term “selumetinib” OR “AZD6244” OR “AZD 6244” OR “ARRY142886” OR “ARRY 142886” OR “ARRY-142886” was used for the initial search on PubMed, followed by manual selection with the goal of identifying all NSCLC cell types harboring *KRAS* mutation that were treated with selumetinib. The genetic background of *KRAS* and *LKB1* were confirmed through the COSMIC database (http://cancer.sanger.ac.uk). Wild type *LKB1* was also confirmed through literature search. Selumetinib IC50 values were extracted and used for initial study of the correlation between *LKB1* status and sensitivity to selumetinib. The cells were defined as sensitive to selumetinib if their IC50 was equal to or lower than 1uM, or resistant if the IC50 was higher than 1μM as previously described [47].

### Drugs

Selumetinib (AZD6244, Catalog No. S1008) and phenformin (Catalog No. S2542) were purchased from Selleck Chemicals. For *in vitro* studies, selumetinib was prepared as 20mM stock solution in DMSO and phenformin 200 mM in H_2_O. For *in vivo* studies, selumetinib was first dissolved into a homogenous suspension in a minimum volume of DMSO, and then diluted in H_2_O to a final concentration of 20 mg/ml. Phenformin was dissolved in H_2_O with a final concentration of 40 mg/ml. 50 μl of each drug was used for daily oral gavage.

### Culture of cell lines and assessment of cytotoxicity of selumetinib, phenformin, or their combinations

NSCLC cell lines A549, H460 and H157 were purchased from the American Type Culture Collection (ATCC), and their identities were verified by genotyping service at Emory University. Mutations in LKB1 in A549, H460 and H157 were verified by genomic sequencing [48]. The pBABEpuro-based retroviruses encoding wild type *LKB1* were used to infect and establish stable isogenic cell lines for A549, H460 and H157; all have *KRAS* mutation but *LKB1* inactivation. Here, A549^LKB1^, H460^LKB1^ and H157^LKB^ cells have stable expression of wild-type *LKB1* cDNA, whereas A549^pBabe^, H460^pBabe^ and H157^pBabe^ cells only encode empty vector (*LKB1* deficient) [49]. The cells were grown in RPMI medium supplemented with 10% FBS and 1% penicillin/streptomycin and maintained at 37°C in an incubator under an atmosphere containing 5% CO_2_. As previously described [30], the GEM model derived cell lines 634 (*kras*^G12D/wt^/*p53*^-/-^/*lkb1*^wt/wt^) and t2 (*kras*^G12D/wt^/*p53*^-/-^/*lkb1*^-/-^) were cultured in RPMI medium supplemented with 10% FBS and 1% penicillin/streptomycin/glutamine. The cells were routinely screened for the presence of mycoplasma. Cytotoxic effects were determined using the SRB (sulforhodamine B) method as previously described [50]. Briefly, 150μl of cell suspensions containing ~2000 viable cells in logarithmic growth phase were plated into each well of a 96-well flat bottom plate, and incubated overnight before exposure to selumetinib or phenformin or their combination. Cells were prepared in triplicates or quadruplicates. Upon treatment termination, the floating dead cells and their debris were removed and the attached cells were fixed with cold 10% trichloroacetic acid for 30min at 4°C. Cells were then washed with water and stained with 0.4% SRB (Fisher Scientific) for 30 minutes at room temperature, washed again with 1% acetic acid, followed by stain solubilization with 10mM Tris at room temperature on a shaker for 15 minutes. The plates were read on a plate reader (Biotek Synergy MX) using an absorbance wavelength of 565nm, and cell proliferation status was derived from the raw absorbance (OD) data.

### *In vitro* evaluation of the combination effects of selumetinib and phenformin

Synergy was determined for the isogenic cell line A549^pBabe^ and A549^LKB1^. The cells were exposed for 40 hours to selumetinib at 0, 0.3215, 0.625, 1.25, 2.5, 5, 10 and 20 μM, and phenformin at 0, 10, 20, 40, 80, 160, 320 and 640 μM, in all possible combinations. The results of the combined treatment were analyzed according to the isobolographic method of Chou and Talalay [51] by using the Calcusyn software program (Biosoft). The resulting combination index (CI) was used as a quantitative measure of the degree of interaction between the two drugs. A CI equal to 1 denotes additivity, CI greater than 1 antagonism, and CI value less than 1 indicates synergism [51].

### Colony formation assay

As previously described [52], ~200 isogenic A549^pBabe^ and A549^LKB1^ cells were plated in 6-well plates and incubated with different concentrations of selumetinib and phenformin, as well as their various combinations for 2 weeks. Upon treatment termination, the cell colonies were fixed with glutaraldehyde (6% v/v) and stained with crystal violet (0.5% w/v). Triplicates were performed throughout the studies.

### Apoptosis assay

A549^pBabe^ and A549^LKB1^ cells were treated with either diluted DMSO, selumetinib alone, phenformin alone, or their combination as indicated, and cells were collected after 48 hours by trypsinization, washed with cold × 1 phosphate-buffered saline (PBS), and stained with Annexin V-phycoerythrin (PE) and 7-AAD (BD PharMingen) for 15 minutes at room temperature. The apoptotic population of the samples was then measured using a fluorescence-activated cell sorting (FACS) caliber bench-top flow cytometer (Becton Dickinson). FlowJo software (Tree Star) was used for apoptosis analysis.

### Immunoblotting of effector proteins

Depending on the experimental purposes, cells with or without treatment were subjected to immunoblotting through standard Western blot. Briefly, total protein was extracted from cell lysates and concentration determined. ~20 μg protein was subjected to SDS-PAGE, followed by membrane transfer and antibody incubation. Primary antibodies were anti-LKB1 (cat #3047), anti-total and phospho AMPK (cat #2532 and #2535S), anti-total and phospho ERK (cat #9101 and #9102), anti-total and phospho Akt (cat #9271 and #9272), anti-total and phospho S6 (cat #2317 and #4857) from Cell Signaling Technology; anti-Bcl-XL (cat #sc8392) and anti-Bcl-2 (cat #sc509) from Santa Cruz Biotechnology; and anti-β-actin (cat #A5441) from Sigma Aldrich. Secondary HRP conjugated antibodies (anti-mouse, cat # W4021, and anti-rabbit, cat # W4011) were from Promega. The blots were developed using an enhanced chemiluminescence system as described [53].

### Assessment of the selumetinib and phenformin combination *in vivo*

Female athymic nude mice, 4 to 6 weeks old, were used in this study. The animal experimental protocol was approved by the Institutional Animal Care and Use Committees of Emory University. In order to have a direct comparison, ~ 1.5 million A549^pBabe^ and A549^LKB1^ cells were implanted on the left and right flank of the same nude mice respectively. Once the tumors became palpable, mice were orally gavaged daily with either diluted DMSO (Ctrl), selumetinib (S, 50 mg/kg), phenformin (P, 100 mg/kg) or the combination (S+P), 5 days per week. Phenformin solution was prepared directly in water. Since selumetinib does not dissolve well in water, a few drops of DMSO was added initially to the calculated “master” dose just to achieve a homogenous suspension, and then water was added to make the “master” volume, followed by aliquot preparation for daily use. The volume of DMSO used was < 1%. This suspension was either vortexed or finger-tap mixed each time before being administered via oral gavage. The tumor volume and body weight were measured periodically 2~3 times a week. Tumor volume was calculated using the formula: V = 1/2 * AB^2^ where A and B are two perpendicular tumor diameters measured by a caliper, and A > = B. Upon sacrifice, tumors were dissected and weighed, followed by tissue fixation, sectioning and immunohistochemistry (IHC). Major organs such as lungs, liver, spleen, kidney etc. were also harvested for toxicity evaluation.

### Immunohistochemistry staining and analysis

Xenograft tumor tissues were harvested, and then formalin fixed and paraffin embedded before sectioning. First, the sections were pre-heated at 60 degrees for 30 minutes, passed through a series of xylene and alcohol treatments followed by antigen retrieval using 1× citrate buffer for 10 minutes. The slides were allowed to cool for 30 minutes at room temperature and quenched using 3% hydrogen peroxide in distilled water. The sections were washed and then blocked using 2.5% normal horse serum following the instructions from the Kit (Vectastain Kit, Vector Laboratories). Besides H&E staining, standard IHC was used to compare phospho ERK (cat #4370), S6 (cat #4857), as well as Ki67 (prediluted from Life Technologies).

### Statistical analysis

The comparison of mean tumor volume on the growth curve was carried out using one-way ANOVA. The median tumor weights after dissection were compared with nonparametric Kruskal–Wallis test, and presented using Box-and-Whisker plots. For comparison between two groups, student t test was used to compare the means whereas the Mann–Whitney test was used to compare the median. SPSS version 20 was used to conduct these statistical calculations. All *P* values were two-sided and values less than 0.05 were considered statistically significant.

## SUPPLEMENTARY MATERIALS FIGURES AND TABLE



## Abbreviations

AMPK: AMP-activate protein kinase
ERK: extracellular-signal-regulated kinase
GEM: genetically engineered mouse
LKB1: liver kinase B1
MAPK: mitogen-activated protein kinase
MEF: mouse embryonic fibroblast
MEK: MAPK/ERK kinase
MEKi: MAPK/ERK kinase inhibitor
mTOR: mammalian target of rapamycin
NSCLC: non-small cell lung cancer
