# Supplementary material for: Phenformin enhances the therapeutic effect of selumetinib in KRAS-mutant non-small cell lung cancer irrespective of LKB1 status
Source: Oncotarget. 2017 Aug 1;8(35):59008–22. doi: 10.18632/oncotarget.19779 (PMC5601710; doi:10.18632/oncotarget.19779)
Supplement: Supplementary file 1 [file oncotarget-08-59008-s001.pdf]

Phenformin enhances the therapeutic effect of selumetinib in KRAS-mutant non-small cell lung cancer irrespective of LKB1 status

SUPPLEMENTARY MATERIALS

A

	Sensitive to selumetinib (IC50 <= 1μM)	Resistant to selumetinib (IC50 > 1μM)	In total
LKB1 WT	8	2	10
LKB1 mutant	4	12	16
	12	14	26
Fisher's exact test: p = 0.0138 (two-tailed)			

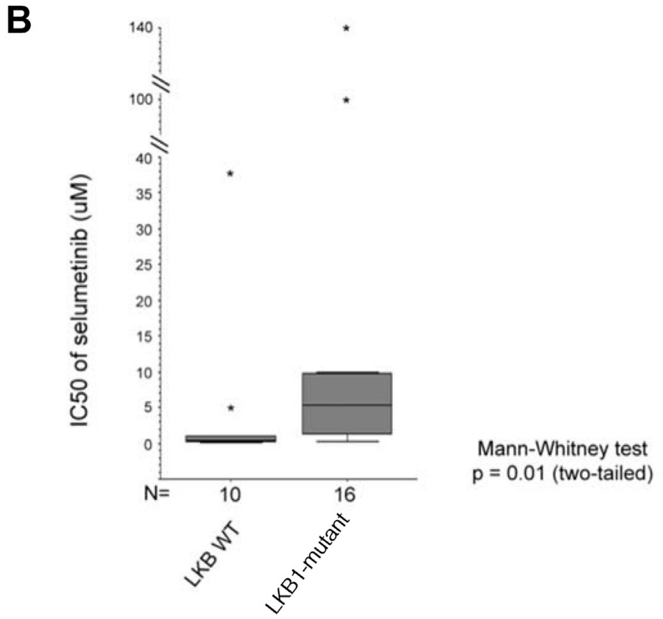

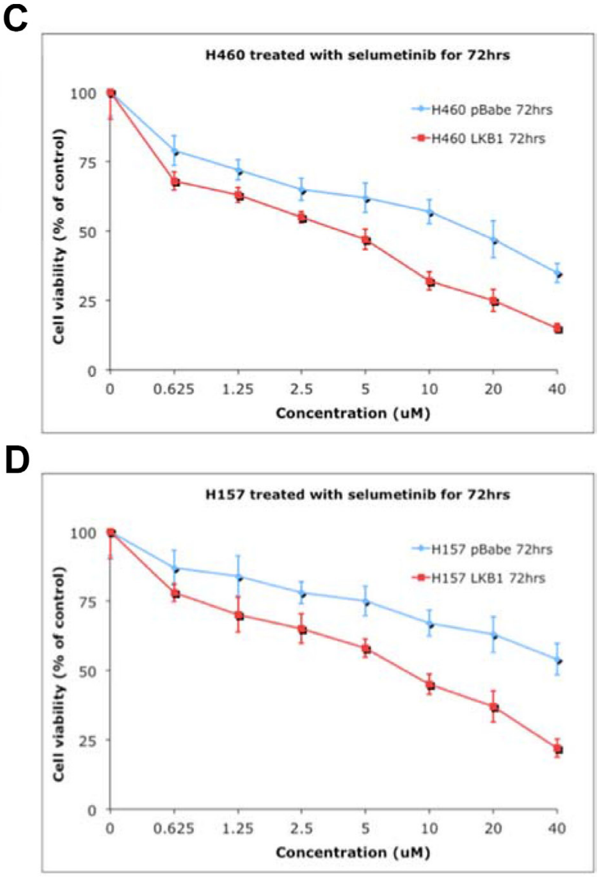

Supplementary Figure 1: Supplemental Figure 1: Concomitant LKB1 mutation correlates with selumetinib resistance in NSCLC cell lines harboring RAS/RAF mutations. Except three cell lines (Calu-1, H358 and H1299) with controversial reported sensitivity to selumetinib, all other cell lines listed in Table 1 are included here for statistical analysis. (**A**) when using IC50 > 1 uM to define resistance to selumetinib, cell lines with concomitant LKB1 mutation (excluding silent mutation) have significantly higher chance of resistance (Fisher's exact test: $p = 0.0138$, two-tailed). (**B**) A direct comparison of IC50 between LKB1 wild type (including silent mutation) and mutant NSCLC cell lines. Whenever possible, for each cell line, the median value of reported IC50 was used for the Mann-Whitney nonparametric test. For cell lines only having a range of value, such as > x or < y uM, then x or y value was used for estimation ($p = 0.01$, two-tailed). (**C** and **D**) Growth inhibition assay of isogenic H460 and H157 cells. The isogenic cells with or without wild type LKB1 were incubated with different concentrations of selumetinib for 72 hrs. With the re-expression of LKB1, cells were more sensitive to selumetinib with lower IC50.

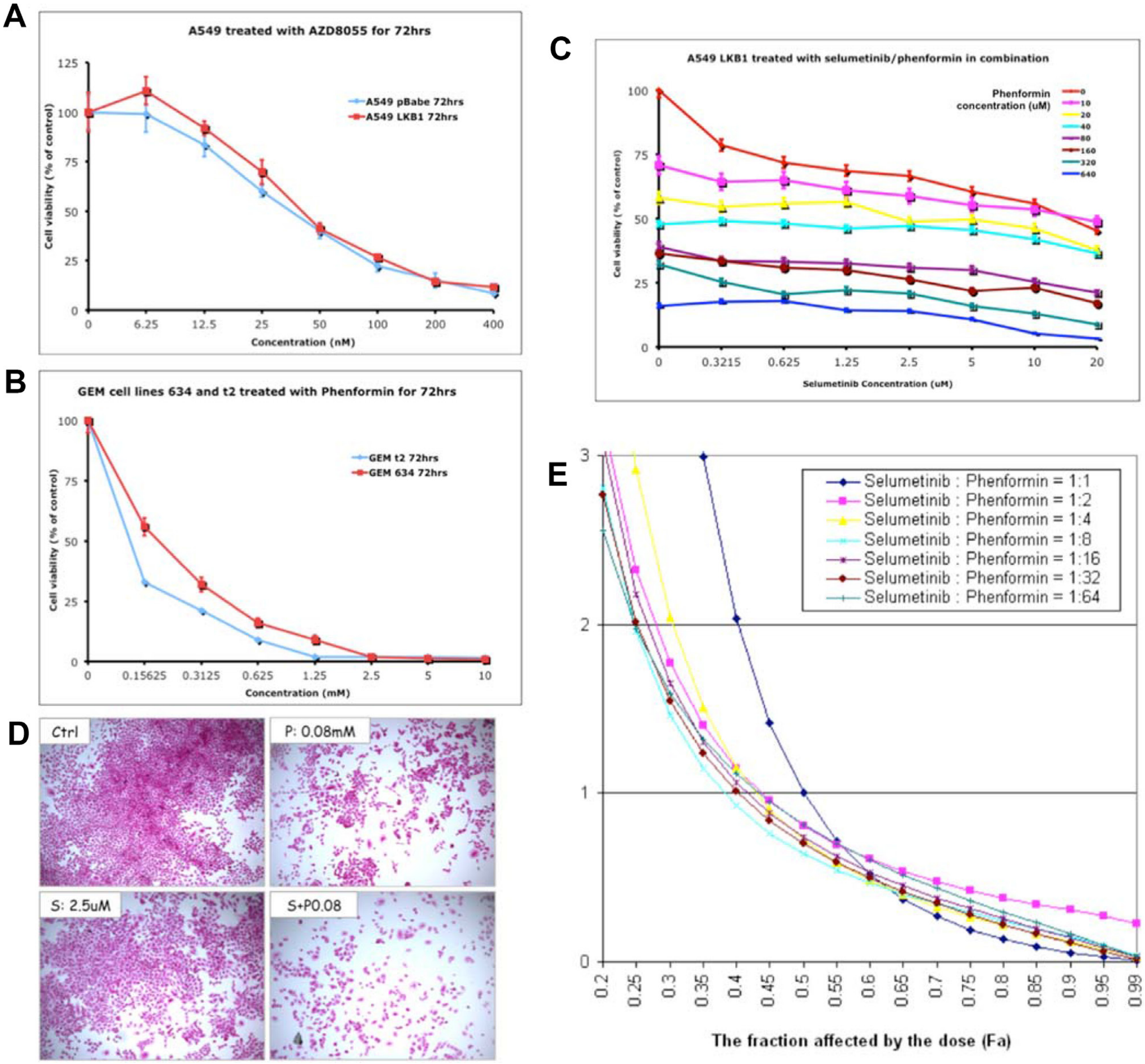

Supplementary Figure 2: LKB1 inactivation dictates enhanced sensitivity to the metabolic drug phenformin, which enhances the activity of selumetinib in KRAS-mutant NSCLC cell lines. (**A**) When the dual mTORC1 and mTORC2 inhibitor AZD8055 was used, no significant difference was observed between the $A549^{pBabe}$ and $A549^{LKB1}$ cells. (**B**) In GEM model-derived cell lines, LKB1 null cells (t2: $kras^{G12D/wt}/p53^{-/-}/lkb1$-/-) were more sensitive to phenformin than those with wild type LKB1 (634: $kras^{G12D/wt}/p53^{-/-}/lkb1^{wt/wt}$). (**C**) Phenformin also enhanced selumetinib activity in $A549^{LKB1}$ cells. Shown here is cell proliferation assay using different concentration and ratio of selumetinib and phenformin in combination. The experiment ended at ~ 40 hrs after incubation. Cells were prepared in triplicate. (**D**) An illustration showing $A549^{pBabe}$ cells treated for 40hrs with either selumetinib at 2.5 μM, or phenformin 80 μM, or their combination. Similar results were observed in $A549^{LKB1}$ cells (not shown). (**E**) CalcuSyn was used to calculate the combination index. Phenformin could also synergize selumetinib in $A549^{LKB1}$ cells, although the optimal ratio was different from that in $A549^{pBabe}$ cells.

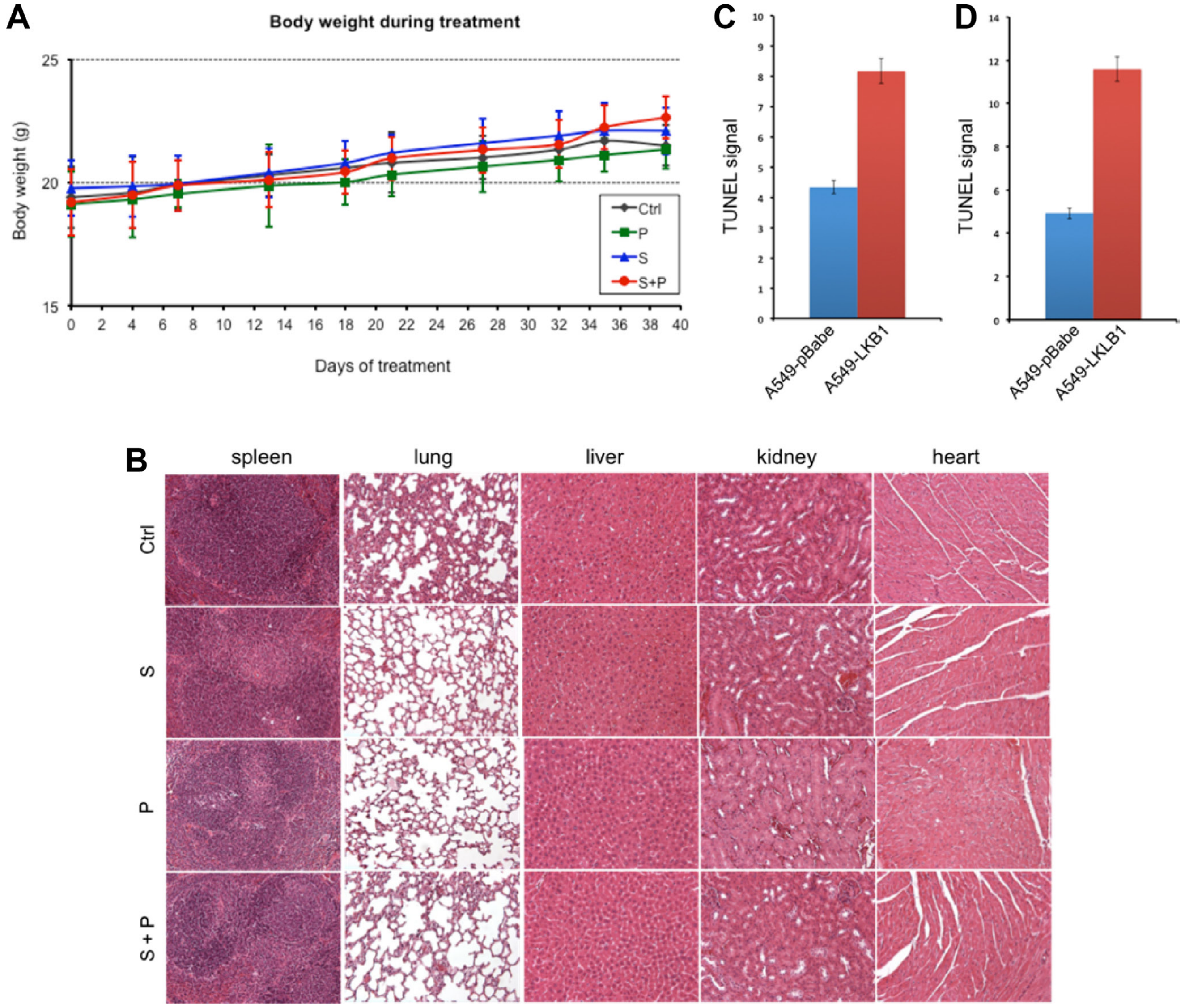

Supplementary Figure 3: Combination treatment with selumetinib and phenformin *in vivo* did not result in significant side effects. (**A**) A comparison of body weight among different groups. There was no significant weight difference observed. (**B**) Microscopic view of the major organs from those treated with combination therapy. Shown here are representative sections stained with H&E from different groups. No obvious toxicity was observed. (**C**) Comparison of matched tumors in the control group. Tumors derived from A549^{LKB1} cells had stronger TUNEL signal, confirming the tumor suppressing function of LKB1. (**D**) Comparison of matched tumors in the "selumetinib only" group. Tumors derived from A549^{pbabe} cells had much weaker TUNEL signal, consistent with the observation that loss of LKB1 confers resistance to selumetinib.

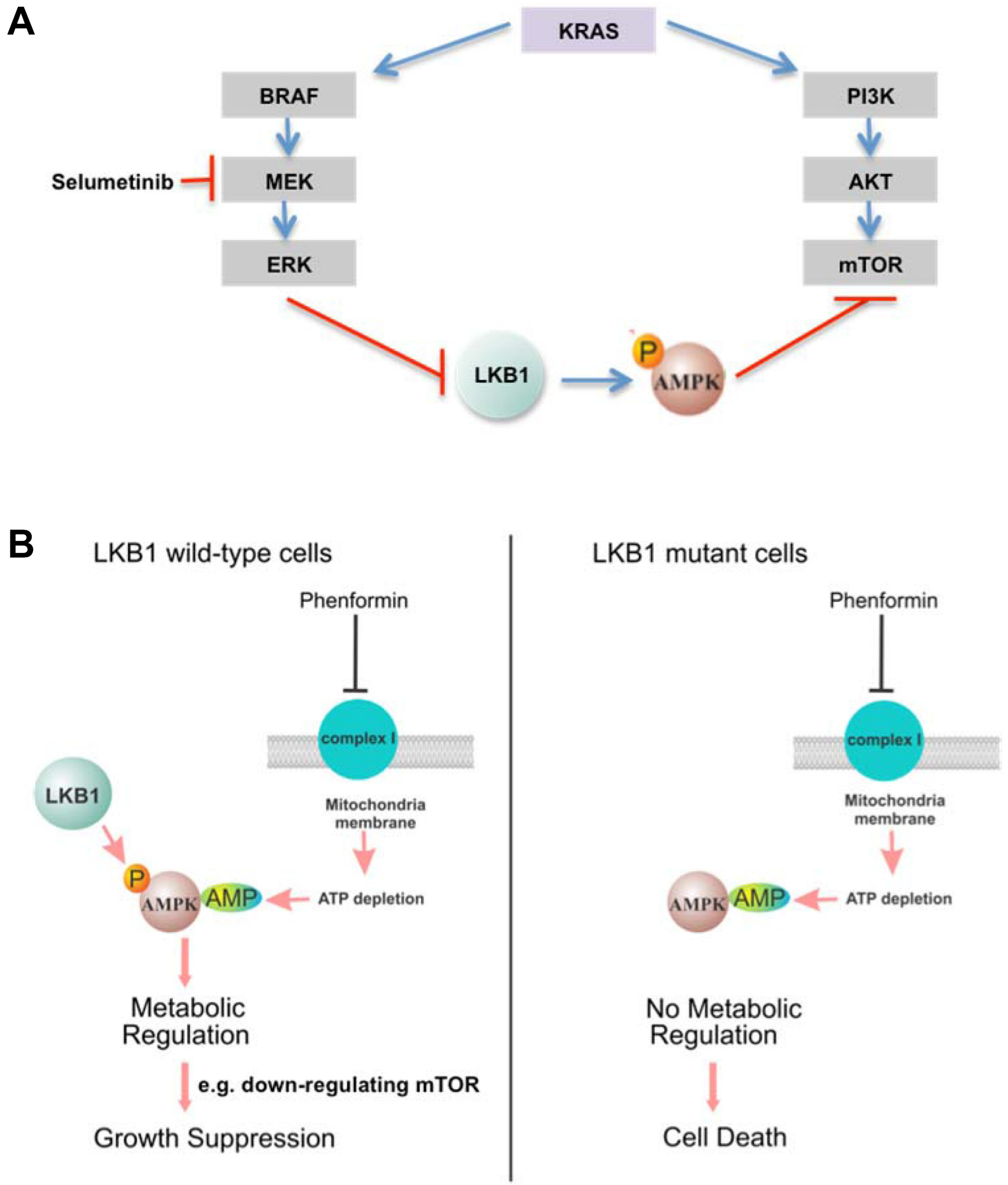

Supplementary Figure 4: A schematic illustration of current project. (**A**) Simplified downstream signaling of KRAS activation. Only BRAF->MEK->ERK and PI3K->AKT->mTOR are shown here. ERK has been reported to induce inhibitory phosphorylation of LKB1[26, 27]. However, it is still not clear whether there is a feed-forward loop from LKB1 to ERK, although others [28] and we did observe LKB1 inactivation results in attenuated p-ERK (Figure 1). This attenuated p-ERK is suggestive of reduced dependency on MEK->ERK signaling pathway, and we speculated it might be the reason of resistance to MEK inhibitor selumetinib in KRAS mutant NSCLC cells harboring LKB1 mutation. Due to escape mechanism, MEK inhibition will normally activate mTOR signaling, warranting co-inhibition of mTOR to achieve maximal inhibitory effect. (**B**) This shows the function of phenformin under alternative LKB1 status. Phenformin targets mitochondria complex 1 and induces ATP depletion. When there is intact LKB1, a metabolic regulation through AMPK activation will ensue – this normally downregulates mTOR signaling and induces growth suppression, hence there is value to combine with MEK inhibition. In contrast, when LKB1 is mutated or inactivated, cells go directly to apoptosis due to the lack of functional metabolic regulation. In fact, others [11] and we have shown LKB1 inactivation dictates the sensitivity to phenformin in NSCLC cells (Figure 2). Based on these rationales, we proposed the combination of selumetinib and phenformin, and have demonstrated their potential synergy that is irrespective of LKB1 status.

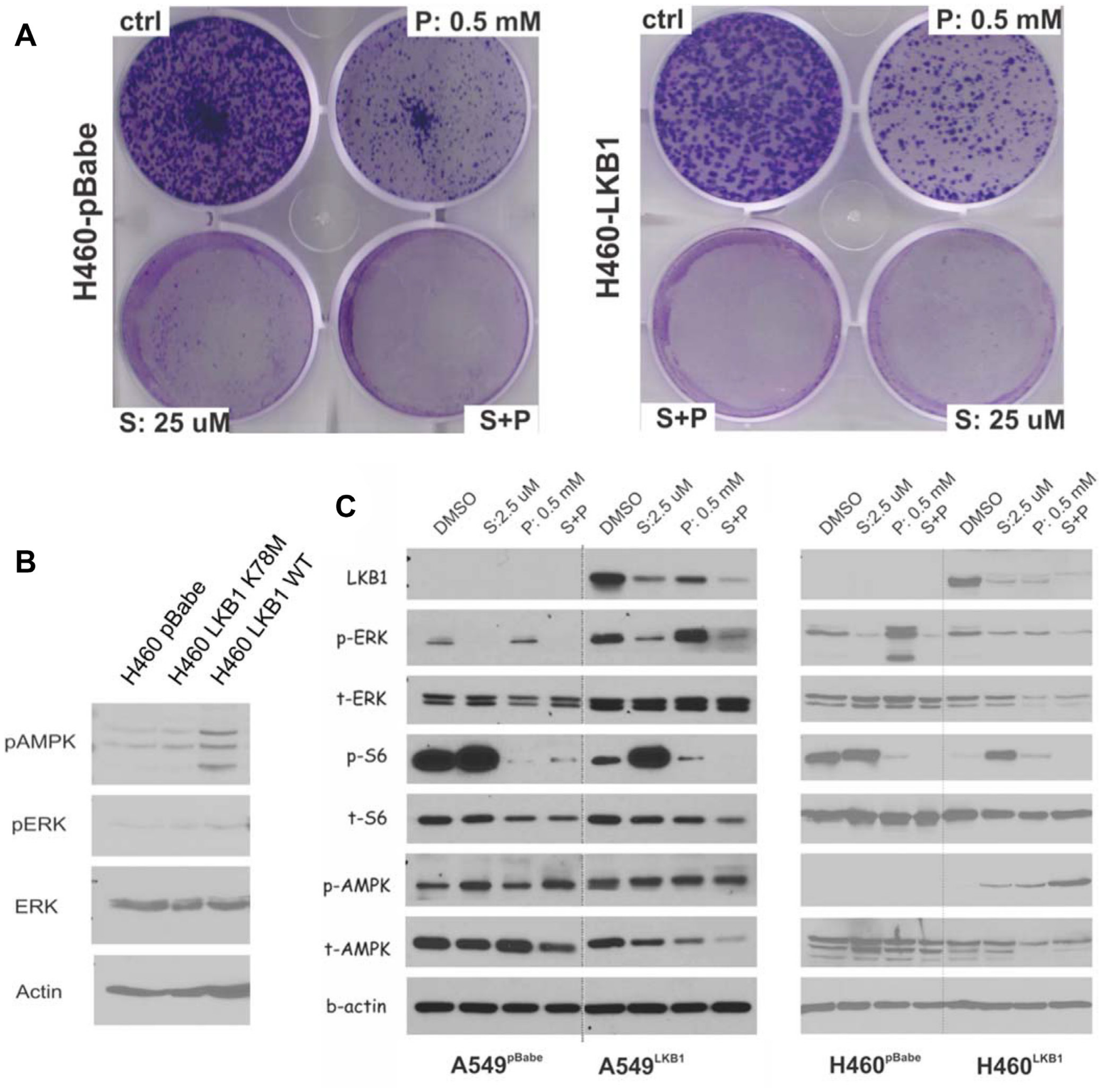

Supplementary Figure 5: Studies using H460 isogenic cells. (**A**) Colony assay of H460 isogenic cells. Although the concentration of selumetinib, as well as the combination ratio of selumetinib and phenformin can be further optimized, it clearly demonstrated that, regardless of LKB1 status, phenformin enhances the therapeutic effect of selumetinib. (**B**) A representative blot to check p-ERK level in H460 isogenic cells. Similar to Figure 1f, the isogenic cells expressing wild type LKB1 exhibit strongest p-ERK level. (**C**) A direct comparison using A549 and H460 isogenic cells. The experimental conditions are identical. Again, in both $H460^{pBabe}$ and $H460^{LKB1}$ cells, the combination of selumetinib and phenformin significantly inhibited the levels of phosphorylated ERK and S6. However, only in $H460^{LKB1}$ cells, such inhibition from combination treatment is parallel to the activation of AMPK.

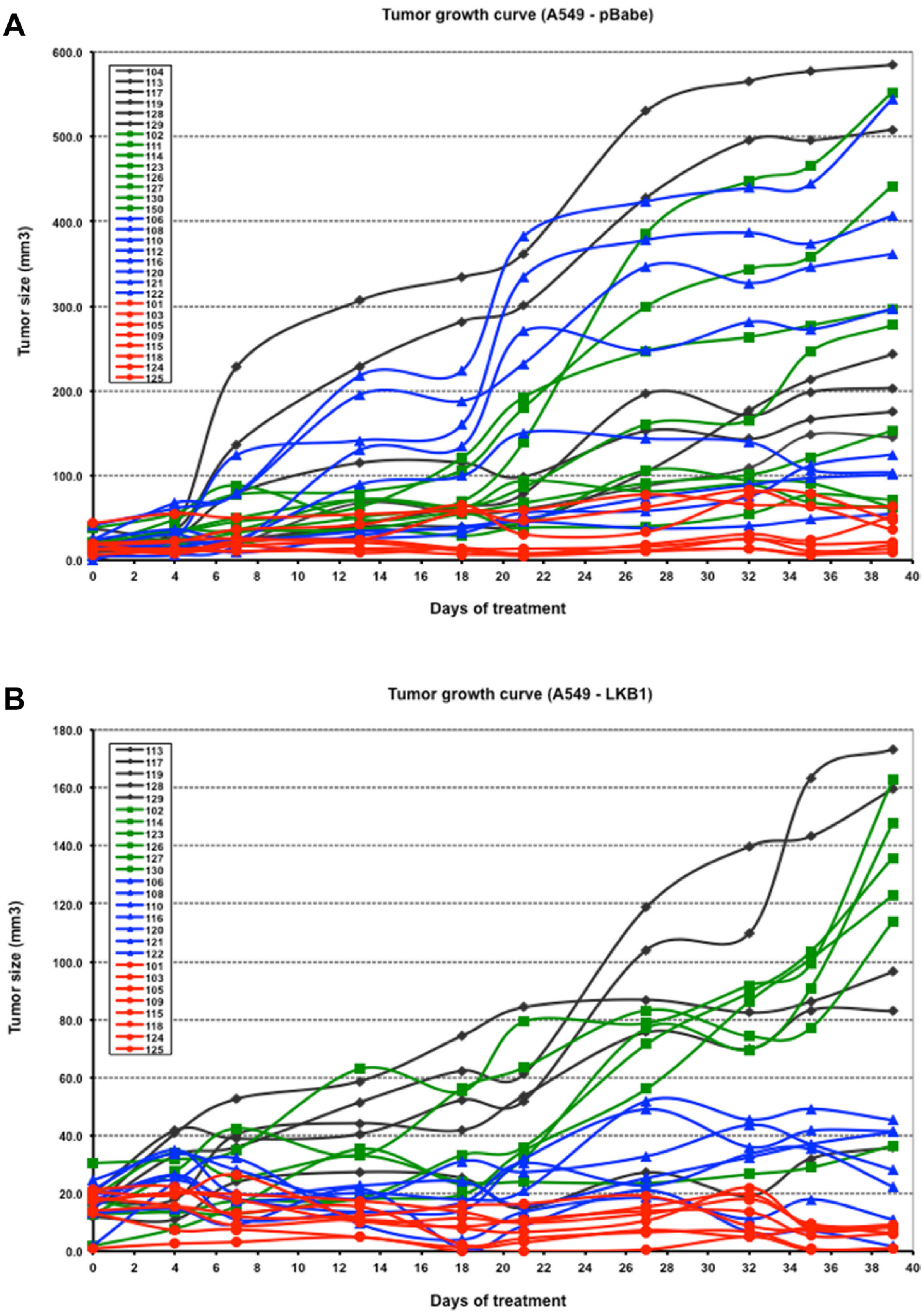

Supplementary Figure 6: Individual growth curves of tumors derived from A549^{pBabe} and A549^{LKB1} cells. Same color scheme is used as in Figure 4b. Black: ctrl; green: phenformin (P) only; blue: selumetinib (S) only; red: P+S. The numbers represent each individual mouse, same as shown in Figure 4c. (**A**) growth curves of tumors derived from A549^{pBabe} cells; (**B**) growth curves of tumors derived from A549^{LKB1} cells. Please notice that the Y-axis scale is different. In addition, mice #112, 150 and 111 were not included in b due to no identifiable tumors.

Supplemental Table 1: Characterization of the 29 NSCLC cell lines with RAS/RAF mutations that were used in the systematic review

Cell line	RAS/RAF status	LKB1 status	Selumetinib IC50 (μM)
Considered as sensitive to selumetinib (IC50 < = 1 μM)			
H441	KRAS G12V	WT[1]	< 0.30[2]
Calu-6	KRAS Q61K	WT[3]	0.32[4], 0.33[2], 1.0[5]
SK-LU-1	KRAS G12D	WT[3]	0.5[5]
H2009	KRAS G12A	WT[6, 7]	0.99[4]
H727	KRAS G12V	WT[1]	0.01[8]
SW900	KRAS G12V	WT[9]	0.28[8]
H1944	KRAS G13D	K62N, K78N	< 0.30[2]
A427	KRAS G12D	Null[10, 11]	0.55[2]
H2122	KRAS G12C	P281fs*6, deletion[10, 12]	< 0.1[5], 1.0[13]
H2347	NRAS Q61R	WT[10]	0.31[4], 0.5[14], < 1[15]
H2087	NRAS Q61K;<br>BRAF L597V	WT[6, 16]	< 0.1[14]
H1666	BRAF G466V	V236fs*30	0.36[8]
Considered as resistant to selumetinib (IC50 > 1 μM)			
A549	KRAS G12S	Q37*	0.8[2], > 1[17], 5[18], ~5[15], 6.3[8], ~10[5], >10[19]
H23	KRAS G12C	W332*	1.5[2], >10[19]
H460	KRAS G12S	Q37*	1.7[2], 9.6[8], > 10[18], > 10[5]
H2030	KRAS G12C	E317*, E357K, M392I	2.2[2]
H2122	KRAS G12C	P281fs*6	~3[15]
H1734	KRAS G13C	M51fs*14	4.2[2]
H157	KRAS G12R	Null[10, 11]	9.3[8], > 10[19]
HCC44	KRAS G12C	M51I, 52 → 162 stop[10]	~10[15]
H1355	KRAS G13C	R49L[6, 20]	~100[15]
H647	KRAS G13D	Null[21]	> 5.0[2], > 10[5]
H2887	KRAS G12V [6, 22, 23]	WT[6]	38[15]
H1155	KRAS Q61H	WT (silent: I46I, P281P)	> 5.0[2]
H1395	BRAF G469A	E57fs*7	> 140[15]
H1755	BRAF G469A	P281fs*6	8.1[8]
Controversial results in literature			
Calu-1	KRAS G12C	WT[12]	< 0.2[2], > 1[17], ~130[15]
H358	KRAS G12C	WT[12]	0.2[19], 0.5[2], 1.0[17], ~10[15], > 10[8]
H1299	NRAS Q61K	WT[24, 25]	< 0.01[18], ~0.3[2], < 0.5[14], > 10[5], > 10[8], > 120[15]

They all have mutations of either the RAS or RAF genes, and were tested with selumetinib. Unless specifically noted, all mutation profiles were confirmed in COSMIC database. Since COSMIC database does not report wild type (WT) genes, the wild type LKB1 status was confirmed through literature search. Numbers in parentheses correspond to the cited study.

REFERENCES

1. Molina-Arcas M, Hancock DC, Sheridan C, Kumar MS, Downward J. Coordinate direct input of both KRAS and IGF1 receptor to activation of PI3 kinase in KRAS-mutant lung cancer. Cancer Discov. 2013; 3:548–563.
2. Garon EB, Finn RS, Hosmer W, Dering J, Ginther C, Adhami S, Kamranpour N, Pitts S, Desai A, Elashoff D, French T, Smith P, Slamon DJ. Identification of common predictive markers of *in vitro* response to the Mek inhibitor selumetinib (AZD6244; ARRY-142886) in human breast cancer and non-small cell lung cancer cell lines. Mol Cancer Ther. 2010; 9:1985–1994.
3. Onozato R, Kosaka T, Achiwa H, Kuwano H, Takahashi T, Yatabe Y, Mitsudomi T. LKB1 gene mutations in Japanese lung cancer patients. Cancer Sci. 2007; 98:1747–1751.
4. Meng J, Peng H, Dai B, Guo W, Wang L, Ji L, Minna JD, Chresta CM, Smith PD, Fang B, Roth JA. High level of AKT activity is associated with resistance to MEK inhibitor AZD6244 (ARRY-142886). Cancer Biol Ther. 2009; 8:2073–2080.
5. Dry JR, Pavey S, Pratilas CA, Harbron C, Runswick S, Hodgson D, Chresta C, McCormack R, Byrne N, Cockerill M, Graham A, Beran G, Cassidy A, Haggerty C, Brown H, Ellison G, et al. Transcriptional pathway signatures predict MEK addiction and response to selumetinib (AZD6244). Cancer Res. 2010; 70:2264–2273.
6. Greer RM, Peyton M, Larsen JE, Girard L, Xie Y, Gazdar AF, Harran P, Wang L, Brekken RA, Wang X, Minna JD. SMAC mimetic (JP1201) sensitizes non-small cell lung cancers to multiple chemotherapy agents in an IAP-dependent but TNF-α-independent manner. Cancer Res. 2011; 71:7640–7648.
7. Mahoney CL, Choudhury B, Davies H, Edkins S, Greenman C, Haaften G, Mironenko T, Santarius T, Stevens C, Stratton MR, Futreal PA. LKB1/KRAS mutant lung cancers constitute a genetic subset of NSCLC with increased sensitivity to MAPK and mTOR signalling inhibition. Br J Cancer. 2009; 100:370–375.
8. Friday BB, Yu C, Dy GK, Smith PD, Wang L, Thibodeau SN, Adjei AA. BRAF V600E disrupts AZD6244-induced abrogation of negative feedback pathways between extracellular signal-regulated kinase and Raf proteins. Cancer Res. 2008; 68:6145–6153.
9. Hata AN, Yeo A, Faber AC, Lifshits E, Chen Z, Cheng KA, Walton Z, Sarosiek KA, Letai A, Heist RS, Mino-Kenudson M, Wong KK, Engelman JA. Failure to induce apoptosis via BCL-2 family proteins underlies lack of efficacy of combined MEK, PI3K inhibitors for KRAS mutant lung cancers. Cancer Res. 2014; 74:3146–56.
10. Matsumoto S, Iwakawa R, Takahashi K, Kohno T, Nakanishi Y, Matsuno Y, Suzuki K, Nakamoto M, Shimizu E, Minna JD, Yokota J. Prevalence and specificity of LKB1 genetic alterations in lung cancers. Oncogene. 2007; 26:5911–5918.
11. Shackelford DB, Abt E, Gerken L, Vasquez DS, Seki A, Leblanc M, Wei L, Fishbein MC, Czernin J, Mischel PS, Shaw RJ. LKB1 inactivation dictates therapeutic response of non-small cell lung cancer to the metabolism drug phenformin. Cancer Cell. 2013; 23:143–158.
12. Liu Y, Marks K, Cowley GS, Carretero J, Liu Q, Nieland TJ, Xu C, Cohoon TJ, Gao P, Zhang Y, Chen Z, Altabef AB, Tchaicha JH, Wang X, Choe S, Driggers EM, et al. Metabolic and functional genomic studies identify deoxythymidylate kinase as a target in LKB1-mutant lung cancer. Cancer Discov. 2013; 3:870–879.
13. Sun C, Hobor S, Bertotti A, Zecchin D, Huang S, Galimi F, Cottino F, Prahallad A, Grernrum W, Tzani A, Schlicker A, Wessels LF, Smit EF, Thunnissen E, Halonen P, Lieftink C, et al. Intrinsic resistance to MEK inhibition in KRAS mutant lung and colon cancer through transcriptional induction of ERBB3. Cell Rep. 2014; 7:86–93.
14. Ohashi K, Sequist LV, Arcila ME, Lovly CM, Chen X, Rudin CM, Moran T, Camidge DR, Vnencak-Jones CL, Berry L, Pan Y, Sasaki H, Engelman JA, Garon EB, Dubinett SM, Franklin WA, et al. Characteristics of lung cancers harboring NRAS mutations. Clin Cancer Res. 2013; 19:2584–2591.
15. Meng J, Dai B, Fang B, Bekele BN, Bornmann WG, Sun D, Peng Z, Herbst RS, Papadimitrakopoulou V, Minna JD, Peyton M, Roth JA. Combination treatment with MEK, AKT inhibitors is more effective than each drug alone in human non-small cell lung cancer in vitro and in vivo. PloS one. 2010; 5(11):e14124.
16. Komiya T, Coxon A, Park Y, Chen WD, Zajac-Kaye M, Meltzer P, Karpova T, Kaye FJ. Enhanced activity of the CREB co-activator Crtc1 in LKB1 null lung cancer. Oncogene. 2010; 29:1672–1680.
17. Acquaviva J, Smith DL, Sang J, Friedland JC, He S, Sequeira M, Zhang C, Wada Y, Proia DA. Targeting KRAS-mutant non-small cell lung cancer with the Hsp90 inhibitor ganetespib. Mol Cancer Ther. 2012; 11:2633–2643.
18. Troiani T, Vecchione L, Martinelli E, Capasso A, Costantino S, Ciuffreda LP, Morgillo F, Vitagliano D, D'Aiuto E, De Palma R, Tejpar S, Van Cutsem E, De Lorenzi M, Caraglia M, Berrino L, Ciardiello F. Intrinsic resistance to selumetinib, a selective inhibitor of MEK1/2, by cAMP-dependent protein kinase A activation in human lung and colorectal cancer cells. Br J Cancer. 2012; 106:1648–1659.
19. Yoon YK, Kim HP, Han SW, Oh dY, Im SA, Bang YJ, Kim TY. KRAS mutant lung cancer cells are differentially responsive to MEK inhibitor due to AKT or STAT3 activation: implication for combinatorial approach. Mol Carcinog. 2010; 49:353–362.
20. Tsai LH, Chen PM, Cheng YW, Chen CY, Sheu GT, Wu TC, Lee H. LKB1 loss by alteration of the NKX2-1/p53 pathway promotes tumor malignancy and predicts poor survival and relapse in lung adenocarcinomas. Oncogene. 2014;33:3851–3860.

21. Wallin JJ, Edgar KA, Guan J, Berry M, Prior WW, Lee L, Lesnick JD, Lewis C, Nonomiya J, Pang J, Salphati L, Olivero AG, Sutherlin DP, O'Brien C, Spoerke JM, Patel S, et al. GDC-0980 is a novel class I PI3K/mTOR kinase inhibitor with robust activity in cancer models driven by the PI3K pathway. Mol Cancer Ther. 2011; 10:2426–2436.

22. Querings S, Altmüller J, Ansén S, Zander T, Seidel D, Gabler F, Peifer M, Markert E, Stemshorn K, Timmermann B, Saal B, Klose S, Ernestus K, Scheffler M, Engel-Riedel W, Stoelben E, et al. Benchmarking of mutation diagnostics in clinical lung cancer specimens. PloS one. 2011; 6:e19601.

23. Barbie DA, Tamayo P, Boehm JS, Kim SY, Moody SE, Dunn IF, Schinzel AC, Sandy P, Meylan E, Scholl C, Fröhling S, Chan EM, Sos ML, Michel K, Mermel C, Silver SJ, et al. Systematic RNA interference reveals that oncogenic KRAS-driven cancers require TBK1. Nature. 2009; 462:108–112.

24. Zhong D, Liu X, Khuri FR, Sun SY, Vertino PM, Zhou W. LKB1 is necessary for Akt-mediated phosphorylation of proapoptotic proteins. Cancer Res. 2008; 68:7270–7277.

25. Zhang S, Schafer-Hales K, Khuri FR, Zhou W, Vertino PM, Marcus AI. The tumor suppressor LKB1 regulates lung cancer cell polarity by mediating cdc42 recruitment and activity. Cancer Res. 2008; 68:740–748.

26. Mihaylova MM, Shaw RJ. The AMPK signalling pathway coordinates cell growth, autophagy and metabolism. Nat Cell Biol. 2011; 13:1016–1023.

27. Zheng B, Jeong JH, Asara JM, Yuan YY, Granter SR, Chin L, Cantley LC. Oncogenic B-RAF negatively regulates the tumor suppressor LKB1 to promote melanoma cell proliferation. Mol Cell. 2009; 33:237–247.

28. Chen Z, Cheng K, Walton Z, Wang Y, Ebi H, Shimamura T, Liu Y, Tupper T, Ouyang J, Li J, Gao P, Woo MS, Xu C, Yanagita M, Altabef A, Wang S, et al. A murine lung cancer co-clinical trial identifies genetic modifiers of therapeutic response. Nature. 2012; 483:613–617.
